# Cognitive constraints on vocal combinatoriality in a social bird

**DOI:** 10.1016/j.isci.2023.106977

**Published:** 2023-05-26

**Authors:** Stuart K. Watson, Joseph G. Mine, Louis G. O’Neill, Jutta L. Mueller, Andrew F. Russell, Simon W. Townsend

**Affiliations:** 1 Department of Comparative Language Science, University of Zurich, Zurich, Switzerland; 2 Department of Evolutionary Biology and Environmental Studies, University of Zurich, Zurich, Switzerland; 3 Center for the Interdisciplinary Study of Language Evolution, Zurich, Switzerland; 4 Faculty of Environment, Science and Economy, University of Exeter, Penryn, Cornwall TR10 9FE, UK; 5 Department of Biological Sciences, Macquarie University, North Ryde, NSW 2109 Australia; 6 Institute of Linguistics, University of Vienna, Vienna, Austria; 7 Fowlers Gap Arid Zone Research Station, School of Biological, Earth & Environmental Sciences, University of New South Wales, Sydney, NSW 2052, Australia; 8 Department of Psychology, University of Warwick, Coventry, UK

**Keywords:** Biological sciences, Evolutionary biology, Evolutionary processes

## Abstract

A critical component of language is the ability to recombine sounds into larger structures. Although animals also reuse sound elements across call combinations to generate meaning, examples are generally limited to pairs of distinct elements, even when repertoires contain sufficient sounds to generate hundreds of combinations. This combinatoriality might be constrained by the perceptual-cognitive demands of disambiguating between complex sound sequences that share elements. We test this hypothesis by probing the capacity of chestnut-crowned babblers to process combinations of two versus three distinct acoustic elements. We found babblers responded quicker and for longer toward playbacks of recombined versus familiar bi-element sequences, but no evidence of differential responses toward playbacks of recombined versus familiar tri-element sequences, suggesting a cognitively prohibitive jump in processing demands. We propose that overcoming constraints in the ability to process increasingly complex combinatorial signals was necessary for the productive combinatoriality that is characteristic of language to emerge.

## Highlights

• The ability to freely recombine a repertoire of distinct elements is key to language
• Chestnut-crowned babblers’ vocal system is limited to combinations of two distinct sounds
• We explored limits of this combinatoriality using an artificial grammar experiment
• Data shows babblers can process recombinations of bi- but not tri-element sequences

## Graphical abstract

**Figure:**
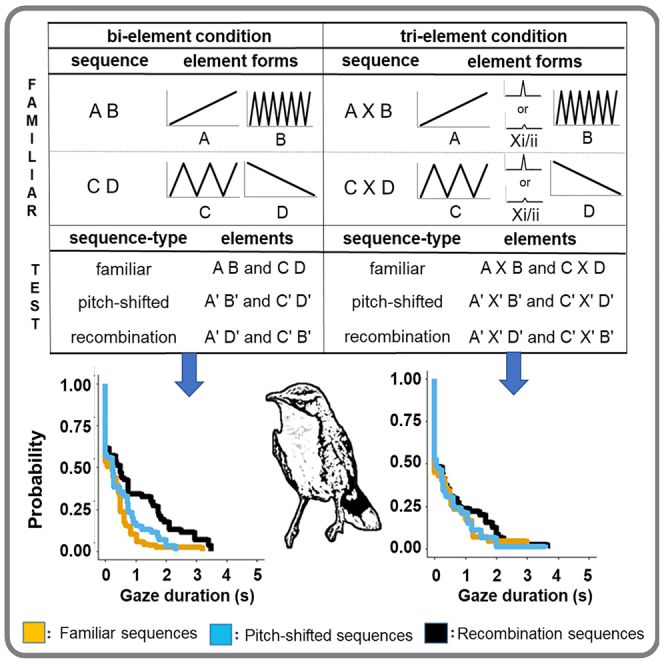

## Introduction

The comparative approach is a powerful tool for examining the evolutionary roots of uniquely human cognitive capacities.^1^ The application of this approach to the study of language evolution has been particularly fruitful, identifying many of the cognitive building blocks necessary (but not sufficient) for the emergence of language in a diverse range of non-human animal communication systems.^2^^,^^3^ Notably, experiments confirm that birds and mammals can change the meaning of their vocalizations by joining calls together in sequences consistent with basic syntax^4^^,^^5^^,^^6^^,^^7^ or by recombining meaningless call elements in different ways to generate alternative meanings in a manner analogous to basic a phonemic system.^8^^,^^9^^,^^10^ However, in stark contrast to the complex combinations of words and phonemes in human languages, animals seldom use the same calls or call elements across meaningful sequences of more than two distinct sound units.^4^^,^^11^^,^^12^^,^^13^^,^^14^ The bi-element structuring of combinatorial calls in animals cannot be due to constraints on vocal production. Many animals have vocal repertoires containing a sufficient number of sounds to theoretically be able to generate hundreds of different sound combinations, since a factorial function underpins the relationship between the number of sounds and the potential number of sound combinations. Indeed, some species of bird can sing hundreds of different songs,^15^ but as far as we know, varying a song's composition does not change the semantic meaning.^16^ However, even songs that appear to be highly complex can be described and processed as a series of bigrams,^17^ and recent evidence suggests that some songbirds may be insensitive to the actual ordering of notes within a song, attending instead to the fine acoustic details of individual notes.^18^ This presents a stark contrast with the processing of meaningful call combinations, where the re-ordering of constituent elements seems to render them unrecognisable in species, such as Japanese tits.^19^ This difference in how the call types seem to be processed, alongside the fact that songs often contain a myriad of elements whereas meaningful calls typically contain just a few, leads to a tantalizing hypothesis: The cognitive demands of processing sequences with fixed positionality increase according to the number of distinct elements within them, therefore placing a cognitive ceiling on the diversity and complexity of combinatorial calls that can exist within a given system.^20^

**Figure 1:**
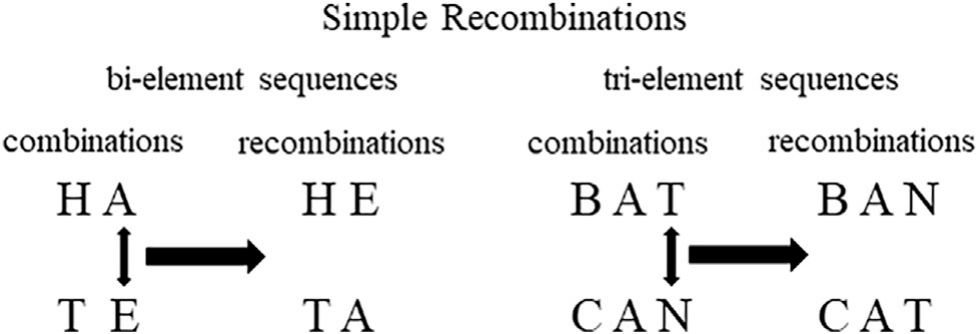
Simple means of generating two new words using reciprocal element recombination in bi- and tri-element sequences Note that in the bi-element condition, processing the sound only requires recognition of the two elements and which one comes first, whereas in the tri-element condition, the sequence cannot be understood without realization of the additional connection between the first and last elements. It is thus anticipated that processing recombinations of tri-element sequences will be more demanding cognitively.

The path to language clearly requires the capacity to generate new meaning by recombining the same sounds across sequences of at least three elements: with just 12 sounds, an order of magnitude more tri-element than bi-element combinations are possible (1320 vs. 132 permutations in each case). However, unlike for bi-element sequences, understanding recombined sequences of three or more elements will more often require the ability to recognize, retain, and process relationships between distinct sounds that are positioned both consecutively and non-consecutively within a sequence.^21^^,^^22^ For example, the phonemes/b/,/æ/,/t/,/k/and/n/can be combined to make the word *bat*, or recombined to make the words *can*, *ban* or *cat* (Figure 1). In all cases, distinguishing between one meaning and another requires an ability, not only to recognize the constituent elements and their order, but also to process the relationship between the first and last elements. Indeed, infants as young as 10 months old have been found to process non-adjacent relationships between vowels,^23^ consonants,^24^ and fricatives^25^ within language (see^26^ for review). Thus, a generative system, which goes beyond simple two-element combinations, is necessarily more cognitively demanding than a more constrained system due to the number of relationships that will often need mapping, and commensurate increases in working-memory requirements.^22^^,^^27^ This is also reflected by the fact that the human capacity to process relationships between non-adjacent elements comes online at a later stage in development than the processing of adjacent ones.^28^^,^^29^ However, it is worth mentioning that not all non-adjacent elements in a string are necessarily processed as such depending on the layer of representation at work.^30^

One way of testing the capacity for animals to process recombinations of sound sequences of contrasting structural complexity is to use an “artificial grammar” approach.^31^^,^^32^ In such experiments, subjects can be familiarized to artificial sequences of two versus three distinct acoustic elements, for example, and then have their ability to process the associations between constituent elements in each case tested through sequence violations.^33^ Using sequences of artificial sounds rather than natural call elements from the subject’s repertoire will invariably be important to remove confounding biases of prior expectation given natural element use.^34^ In addition, using artificial sequences allows one to better generalize perceptual processing capacity across species than does the use of species-specific elements.^33^ Previous studies have used this type of artificial grammar approach to probe the extent to which non-human animals can process the kinds of structures underlying syntactic and phonological structures.^3^^,^^21^^,^^22^^,^^35^^,^^36^ Here, we apply this approach in a species which naturally makes use of a limited variety of meaningful sound combinations, to elucidate the cognitive constraints that limit the productive recombination of meaningless call elements across calls, a prerequisite for productive word generation.

Previously, we have provided evidence to suggest that the highly social chestnut-crowned babbler (*Pomatostomus ruficeps*) of outback south-eastern Australia produces sound combinations with properties superficially analogous to phonemic contrasts found in human language.^8^^,^^9^ This species does not sing and is not known to be a vocal learner, but possesses a repertoire of at least 18 calls (two of which are combinatorial).^37^ In addition, just six of these calls are comprised a single element, while the rest are sequences of 2–5 distinct elements, usually in a stereotyped order. Moreover, we have demonstrated that the same call elements can be used in different combinations across functionally distinct contexts. Specifically, movement is associated with the production of a two-sound element “flight” call of the form “A-B” (where A and B are acoustically distinct elements), while nestling provisioning is associated with “prompt” calls which contain the same two elements, but wherein the B element is repeated to form the sequence “B-A-B.” Experiments have confirmed that the A and B elements produced in flight calls are acoustically and perceptually indistinguishable from those found in prompt calls and are meaningless, in that they do not convey context-specific information.^8^^,^^9^ Yet, this case of combinatoriality is non-productive—i.e. two elements combine to make just two functionally distinct calls, and neither of the sounds is combined in other sequences. Thus, although babblers have a rich call repertoire and can both produce and perceive calls comprising up to five distinct elements, they do not recombine elements across calls of three or more distinct elements. A parsimonious hypothesis is that babblers are constrained to recombine sounds across calls with just two distinct elements because they lack the cognitive capacity to process variation in the composition of sound sequences involving three or more distinct sound elements.^20^

**Figure 2:**
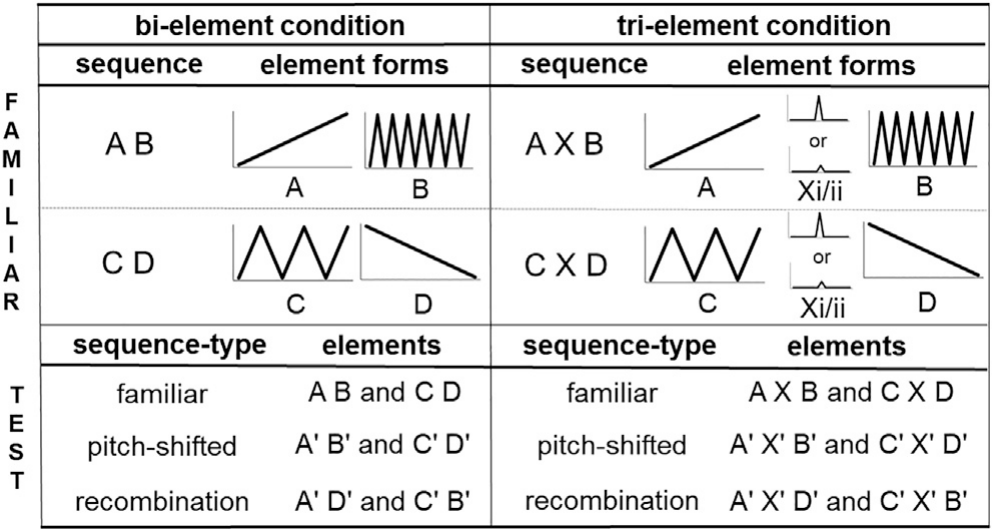
Visual representation of each element type (A-D, Xi, Xii) and how they were combined in each sequence type in the familiarization (top) and test phases (bottom) Familiar elements (e.g. A) could be drawn from any of pitch variants 1–8 of that sound, while pitch-shifted elements (e.g. A′) were drawn from variants 9–16. X elements could be of form either Xi or Xii to minimize associations between central and edge elements. Pitch-shifted sequences were to determine whether any effects observed in response to recombination sequences could be explained by mere acoustic novelty rather than learning of sequences. Audio file examples of each sequence type are available at: https://osf.io/mhgcx/.

Here, we tested the capacity of wild-caught chestnut-crowned babblers to process combinations and recombinations of artificial sounds in sequences of two versus three distinct elements in standardised settings (Figure 2). More specifically, we first familiarized chestnut-crowned babblers to sequences of frequency-modulated sine-tone elements involving two or three distinct element types. We then played them sets of three test trials comprising: (i) the same sound sequences to which they were familiarized (“familiar sequences”); (ii) the same sound sequences but pitch-shifted, to test whether the birds can abstract the familiar categorical associations to novel acoustic stimuli (“pitch-shifted sequences”); and (iii) familiar sounds, but in pitch-shifted combinations that also violated the familiar associations between them, to test whether birds can recognize recombinations of the familiar sequences (“recombination sequences”). Throughout the three types of test trial sequence, we measured the subjects’ latency to look toward the source of the sound, as well as the duration of their gaze. If babblers are able to process a given sequence type, we predicted that they would react similarly to familiar and pitch-shifted sequences (given that they are structured according to the same rule). Recombination sequences, on the other hand, should elicit a stronger response because they are unfamiliar recombinations of sounds.^33^^,^^38^ Given that chestnut-crowned babblers routinely produce calls comprising both two and three distinct elements, but only use the same elements across sequences of two distinct elements,^37^ we predicted a capacity to detect recombinations of two-, but not three-element sequences in our experiment.

## Results

### Comparison of sequence types within bi- and tri-element conditions

**Figure 3:**
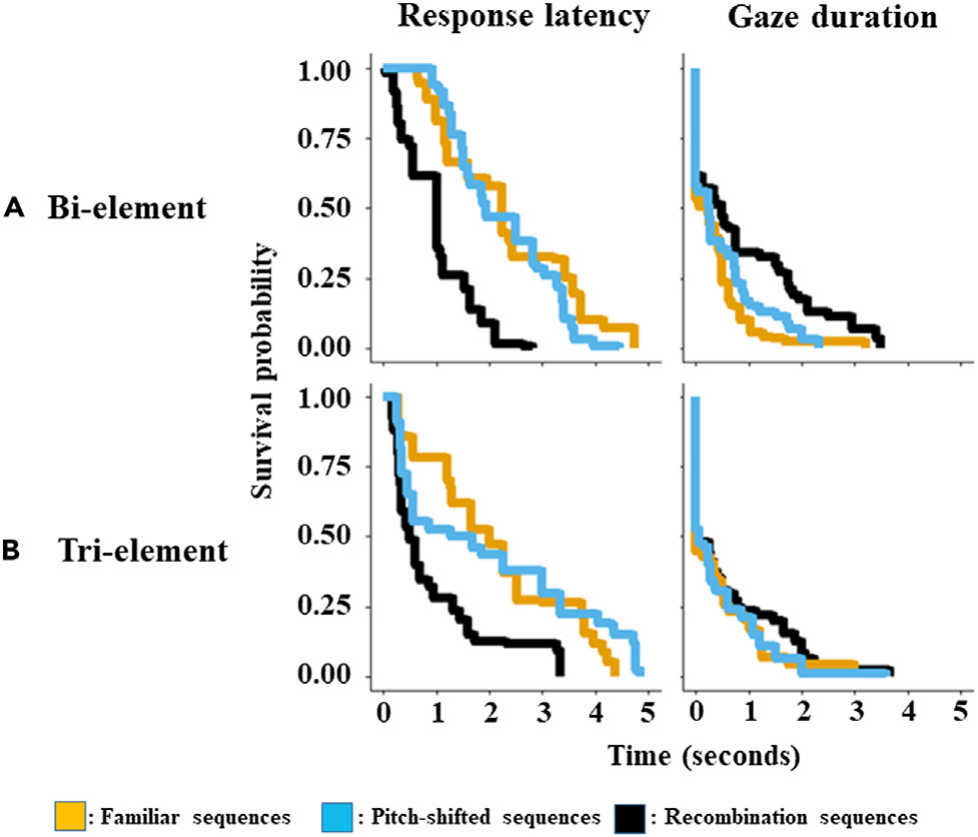
Survival plots for each condition (A: Bi-element, B: Tri-element) and response measure (Top: Latency, bottom: Gaze duration) Lines indicate the probability that a behavior will start (response latency) or stop (gaze duration) after corresponding amounts of time (x axis). Familiar sequences are identical to those heard during the familiarization phase of the experiment. Pitch-shifted sequences are identical in structure to familiar sequences, but individual elements were pitch-shifted to create acoustic novelty. Recombination sequences are unfamiliar recombinations of familiar sound elements, which are also pitch-shifted. Note that while these plots graphically represent the raw data, they do not control for individual identity and repeated measures, unlike the statistical models described in Table 1. For descriptive plots which average data points by individual, see Figure S1.

First, we found evidence to suggest that chestnut-crowned babblers can process the recombinations of artificial sequences comprising two distinct elements. During test playbacks of bi-element sound sequences to which they were familiarized, birds took an average of 2.9 s (±1.79 SD) to look at the speaker and did so for an average of 0.69 s (±0.83 SD) (See Figure S1, Table S1 for full descriptive statistics). Compared to these familiar sequences, latency and gaze duration changed little in response to playbacks where the familiar bi-element sequence orders were pitch-shifted, but both changed substantially toward playbacks of recombination sequences, in which learned element orders were violated (Figure 3, Table 1). Moreover, for both response latency and gaze duration, the full model (which included playback sequence type as a fixed effect and random effects for individual and group) was a substantially better predictive fit for the data than the null model (which included only random effects, Table 1). For example, as indicated by the hazard ratios in Table 1, birds responded to recombination sequences on average 1.44× faster and for 1.92× longer than toward pitch-shifted sequences, as well as 1.54× faster and 2.14× longer relative to familiar sequences (Figure 3, Table 1). From these results, we can infer that the birds were readily able to learn the order of the elements in bi-element sequences, and were furthermore sensitive to unfamiliar recombinations of these elements.

**Table 1:** Outputs for Bayesian survival models comparing behavioral response (latency to look at the speaker and gaze duration) during playbacks of recombination sequences (e.g. AD instead of AB) relative to responses during familiar sequences (e.g. AB) or pitch-shifted variants of familiar sequences

Condition	Response	wAIC weight	Sequence-contrast	Hazard ratio	Hazard ratio lower 95% bound	Hazard ratio upper 95% bound
Bi-element	Response latency	0.75	Recombination vs. pitch-shift Recombination vs. familiar	0.54 0.46	0.31 0.25	0.98 0.83
Bi-element	Gaze duration	0.89	Recombination vs. pitch-shift Recombination vs. familiar	1.92 2.14	1.09 1.13	3.51 4.00
Tri-element	Response latency	0.09	Recombination vs. pitch-shift Recombination vs. familiar	0.90 0.88	0.47 0.48	1.73 1.66
Tri-element	Gaze duration	0.21	Recombination vs. pitch-shift Recombination vs. familiar	1.48 1.44	0.77 0.73	2.83 2.69

wAIC weight shows the probability that a model has the best predictive fit of those tested. Hazard Ratio indicates the relative rate at which the behavior of interest occurs (first response latency, or cessation of gaze). Hazard ratios that overlap with 1.00 indicate a lack of reliable difference between the sequences.

By contrast, however, there was no evidence the birds could process recombinations during the tri-element condition. Here, birds (N = 11) took an average of 3.34 s (±1.91 SD) to respond to the familiarization sequences and did so for 0.50 s (±0.75 SD) (See Figure S1, Table S1) Here, neither response latencies nor durations reliably differed between sequence-types: responses following recombination sequences were not statistically different from those given in response to playback of familiarization or pitch-shifted sequences (Figure 3B, Table 1). As a consequence, for the tri-element condition, the model including playback trial type as a fixed effect (i.e. familiar, pitch-shifted, recombined) did not explain a reliable amount of variation in either response latency or gaze duration, indicating that sequence type was not a useful predictor for either response measure during tri-element playbacks (Table 1). This lack of difference in reactions to different arrangements of the tri-element sequences suggests that the birds were not able to detect and/or process changes in such call structures.

### Comparison of same sequence types between bi-element and tri-element conditions

**Figure 4:**
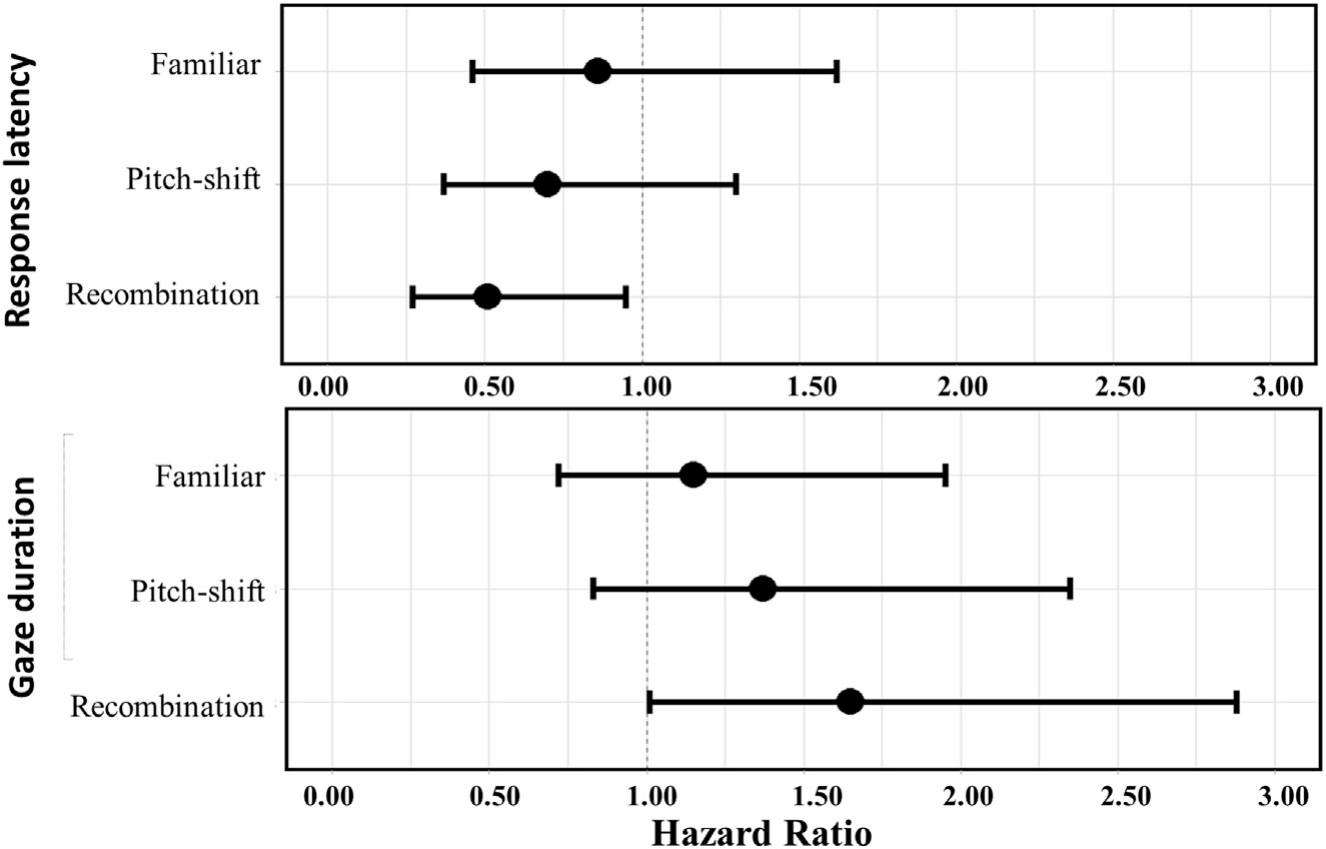
Outputs for between-condition comparisons (bi-element vs. tri-element) of response to sequence types Top: Response latency. Bottom: Gaze duration. Dots represent mean estimate, lines represent 95% credible intervals. 95% CIs that do not overlap with the horizontal line indicate a robust difference between bi- and tri-element conditions.

Finally, to elucidate the reason for the differential responses elicited by different sequence types in the bi- versus tri-element conditions, we compared responses of the same sequence type across the two conditions (e.g. the difference in response to familiarization sequences between bi- and tri-element conditions, see Figure 4). For example, one possibility is that the birds were “confused” by the tri-element condition and became disinterested in the stimuli, but an alternative is that they were able to learn the constituent sounds but not the associations between them sufficiently to process recombinations of those elements. That the latency and duration of responses to familiar sequences were comparable between bi- and tri-element conditions, irrespective of whether or not these sequences were pitch-shifted, suggests that the birds in each condition were similarly familiar with the constituent sounds in the two conditions. However, the response to recombination sequences differed substantially between conditions, with response latency being reduced and gaze duration being extended in bi-element relative to tri-element sequences (Figure 4). These results suggest that the jump from processing recombinations of two versus three distinct elements is cognitively challenging, such that birds were no longer able to keep track of the way in which sequences of three distinct elements were combined and so the critical links between the first and third elements.

## Discussion

Both of our behavioral measures showed that only in the bi-element condition did individuals recognize recombinations of familiar sounds as being distinct from the specific artificial sequences to which they were familiarized. Importantly, in the bi-element condition, responses were substantially stronger during recombination sequences than during playbacks of familiar sequences of pitch-shifted elements, suggesting that increased responses cannot be explained by the acoustic novelty of stimuli *per se*. However, we found no evidence that the birds differentiated between familiar, pitch-shifted and recombination sequences in the tri-element condition. We can therefore infer that not only do babblers readily process novel bi-element sequences of artificial sounds and so the predictive relationships between pairs of constituent elements, but that the computational demands of processing recombinations of tri-element sequences appear to be prohibitive. Together, these results suggest that cognitive constraints on processing limit the capacity for chestnut-crowned babblers to recombine sound elements between calls containing three or more distinct elements.

There are several potential reasons why chestnut-crowned babblers did not recognize and/or process recombinations in the tri-element condition. First, they might have been unmotivated or confused by the trial and lost interest during the familiarization phase. We find this explanation unlikely: chestnut-crowned babblers naturally use bi- and tri-element calls in their repertoire,^37^ and so should not have been preferentially unmotivated to engage in, or been specifically confused by, the tri- versus bi-element familiarization phases. Indeed, the behavioral responses observed were comparable during both the familiar and pitch-shifted trials of bi- and tri-element conditions, suggesting that babblers were equivalently engaged and familiar with the sounds in the two conditions. Alternatively, it might be that the babblers could not process recombinations of tri-element sequences as being “violations” of the sequences experienced during familiarization over the previous day. Further work is required to clarify whether this constraint is genetically imposed or arises through a reduced ability to learn, since babblers do not naturally recombine elements across tri-element calls.^8^^,^^9^ A further possible explanation is that the ecological urgency of B-A-B prompt calls in the babbler system has left them resistant to rearrangements of tri-element sequences of any sounds. This would be an interesting case where broader pattern-recognition capabilities of a species are constrained to the extremely narrow range of ecologically relevant possibilities and might be analogous to data showing that the phonotactic constraints of native English speakers (e.g. the phoneme/ŋ/never occurs in the onset position) interfere with their ability to learn artificial grammars that violate these rules.^39^ However, we find this unlikely in the case of chestnut-crowned babblers, as if a signal were of such high ecological urgency one would expect it to evolve to be as unambiguous and salient as possible—i.e. not combinatorial and/or not sharing elements with other less urgent calls. Regardless of the mechanism of the constraint at work, the take-home suggestion is that chestnut-crowned babblers appear to be constrained cognitively from processing recombined tri-element sequences, which at least in part, might explain why babblers do not possess a more productive combinatorial vocal system.^8^^,^^9^^,^^37^

So what specifically makes the tri-element sequences cognitively demanding to process relative to the bi-element sequences? We identify several alternatives. One straightforward possibility is that limited working memory constrains their ability to mentally represent the entire sequence at once so that the birds simply cannot remember what came at the start of the sequence by the time they get to the end of it. While the birds do have tri-element sequences in their natural repertoire (the “B-A-B” prompt calls), their natural call combinations are composed of very short sounds, whereas our tri-element sequences were 4.5s long in total. Hence, this may be beyond the limits of their working memory, or more simply their motivation to attend to the stimuli for a long time given the lack of ecological relevance. A useful control in further work would therefore be to use stimuli which bear a closer temporal resemblance to their natural calls. However, the birds were able to process the sequences in our bi-element condition which were already 3s. A more likely possibility is that recalling three distinct elements places greater working memory demands than just two, as this requires retrieving three rather than two items from long-term memory and also requires a greater understanding of the positionality of the individual elements. As an illustrative example, the heuristics necessary to recognize the sequence “AXA” (“Contains A” + “A occurs at each edge”) can be relatively simple compared to those necessary for processing “AXB” (“Contains A” + “Contains B” + “A occurs first” + “B occurs last”).^40^^,^^41^ This is consistent with artificial grammar studies in humans which find that relationships between distant elements are easier to process when those elements are perceptually similar^35^ or identical.^42^ Further work with chestnut-crowned babblers could unpack the precise nature of the cognitive constraints in play using additional experimental conditions that probe their ability to process (i) novel tri-element sequences comprising just two distinct elements (i.e. AXA) to determine whether sequences of similar length but simpler composition to those we provided are learnable and (ii) the same sequences with additional middle-elements (e.g. AXXA, AXXXA and so on) to explore whether simpler compositions of greater length can be learned.

The results of this study have at least two more general implications which we hope will inspire future research. First, they provide a theoretical platform from which to test the conundrum of why combinatorial calls, where communicative function varies with changes in sequence structure, are typically limited to recombinations of just two distinct sound entities (elements or calls),^4^^,^^5^^,^^20^ whereas animal songs, despite often comprising tens of semantically meaningless sound elements in myriad ways, signal little more than individual presence and current condition.^43^ We propose that the need to disambiguate meaningful call combinations from one another through holistically processing the type and positionality of individual elements means that the number of distinct elements in a sequence is a significant factor (in addition to e.g. sequence length), which may increase the corresponding cognitive demands. Here, we found that chestnut-crowned babblers were unable to do this with sequences of three distinct elements, which may explain why their natural vocal production system only contains recombinations of two distinct elements. This hypothesis could be further explored by carrying out comparative studies with species known to recombine a greater or fewer number of distinct elements than chestnut-crowned babblers, as well as singing species. Second, by extension, we suggest that increases in processing capacity are an evolutionary prerequisite for increases in the productive power of combinatorial signals, with implications for understanding language evolution. For example, although it has been hypothesised that acoustic constraints in generating new, discriminable sounds were key in promoting the switch to combinatorial structuring during hominin evolution,^44^ it seems likely that overcoming the computational demands associated with processing recombinations of sounds with greater than two entities was an additional fundamental step necessary for productive combinatoriality to emerge. Indeed, it is noteworthy that primates, including squirrel monkeys (*Saimiri sciureus*),^45^ marmosets (*Callithrix jacchus*), and chimpanzees (*Pan troglodytes*) appear capable of processing recombinations of artificial tri-element sound sequences, despite not yet having been demonstrated to use tri-element combinatorial signals in their vocal repertoires.^33^^,^^38^^,^^46^ The fact that these species demonstrate a more sophisticated capacity for combinatorial processing than chestnut-crowned babblers, a species that makes habitual use of combinatorial calls is both striking and puzzling. It may therefore be that the capacity to process complex combinatorial structures is not a domain-specific cognitive trait, but rather an expression of a more generalized capacity for pattern recognition with applications to other domains, such as social cognition and foraging.^47^^,^^48^ This may represent yet another avenue in which increases in social and ecological complexity drive commensurate increases in communicative complexity.^49^^,^^50^^,^^51^^,^^52^^,^^53^^,^^54^ One way to shed more light on these important implications is to apply a similar experimental approach to a range of species with combinatorial calls of varying complexity to explore the extent to which the complexity of their production system corresponds with their perceptual capacities. Using standardized “artificial grammar” experiments offers scope for such cross-species comparisons on the coevolution of the capacity to process and produce combinatorial sequences of varying complexity.

### Limitations of the study

The stimuli used in our experiment were artificial and very different in both sound and duration from the natural vocal repertoire of the babblers: our acoustic elements lasted 1500ms each, whereas individual babbler vocalisations are typically less than 500ms long.^37^ It is therefore conceivable that this introduced working memory demands over and above those imposed by the structural properties of our sequences. While the data from our bi-element condition demonstrates this was not an insurmountable hurdle for the birds, it presents a potential 5 confound for their performance in the tri-element condition. Further work may benefit from including additional conditions that use stimuli more acoustically tailored to the features of the study species, to explore the impact of this factor.

## STAR★Methods

### Key resources table

**Table:** 

REAGENT or RESOURCE	SOURCE	IDENTIFIER
**Deposited data**		

Raw data and analysis scripts	Open Science Framework	https://osf.io/mhgcx/ https://doi.org/10.17605/OSF.IO/MHGCX

**Software and algorithms**		

R statistical programming language	R Project	https://www.r-project.org/
Behavioral Observation Research Interactive Software (‘BORIS’)	Universita Di Torino	https://www.boris.unito.it/

### Resource availability

#### Lead contact

Further information and requests should be directed to and will be fulfilled by the lead contact, Stuart K Watson (swatso88@gmail.com).

#### Materials availability

This study did not generate any new unique reagents.

### Experimental model and study participant details

#### Study site and subjects

The study was conducted at Fowlers Gap Arid Zone Research Station in New South Wales, Australia (141°42′E, 31°06′S). This population has been under long-term investigation since 2004, and details of the study site and population are published elsewhere (e.g.^55^^,^^56^). A total of 24 adults in 10 groups (2–5 per group, comprising 20–44% of the group) were captured in mist nets, selected at random, and transported the few kilometres by vehicle in bird bags to on-site aviary compartments (2 × 2 × 2.5m, see^9^ for further details of housing conditions). It was not possible to discern the sex of the subjects from visual cues. Birds from the same group were housed together, and usually settled and began feeding within 20 min of release into the aviary; birds from different groups were not housed concurrently. Birds were maintained on ∼20 mealworms every 3h and water was provided *ad libitum*. Aviary compartments included natural soil substrate, branches and a nest for roosting. All birds were released back into their original groups successfully within 48 h of initial capture, typically gained 1–2 g mass and were accepted back into the group without retribution.^57^

#### Ethical approval

Ethics approval was provided by UNSW Animal Care and Ethics Committee (06/40A), Macquarie University (2107/025), The University of Exeter, NSW National Parks and Wildlife Service and the Australian Bird and Bat Banding Scheme (3340).

### Method details

#### Playback format

The experiment comprised of a familiarisation phase followed by a test phase; in both cases, stimuli were played on a Braven BRV-X speaker (see below for specific details of stimuli composition in each phase). Starting from at least 1 hour post-settlement in the aviary, birds were provided with 10 familiarisation sessions: 5 on Day 1 and 5 on Day 2, with > 30 min between each session. In each familiarisation session, the birds were played a list of 240 familiarisation sequences (see ‘acoustic stimuli’ below for details of sequence construction) with 2500ms of silence between each sequence to increase the likelihood they would be perceived as separate. Half of the birds (N = 12) received familiarisations sequences of 2 x 2 distinct elements (i.e., A-B and C-D; ‘bi-element condition’; Figure 2) and the other half received familiarisations sequences of 2 x 3 distinct elements (i.e., A-X-B and C-X-D; ‘tri-element condition’; Figure 2), with former sessions lasting 24 min each and the latter lasting 32 min each. The full lists of sequences used for familiarisation and test phases can be accessed at: https://osf.io/mhgcx/.

The test phase was performed on individual birds immediately before release, just after dawn on Day 3 (∼45 h after initial capture). To isolate birds for the test phase, birds were removed from the main aviary on nightfall of Day 2 (using a red-light torch) and roosted over-night individually in a covered wooden box (45 × 20 × 20 cm lbh, one side mesh, contained several perches) at room temperature. Test sessions were preceded by a brief ‘refamiliarisation’ session of 60 different sequences taken from the familiarisation sessions. After two minutes of silence, the experimenter commenced playback of 12 test trials. These 12 trials were comprised of: (i) 4 randomly selected familiar sequences of A-B and C-D (bi-element condition) or A-X-B and C-X-D (tri-element condition); (ii) 4 pitch-shifted sequences of A-X-B and C-X-D, to test whether birds had learned the associations between the elements in familiar sequences, even in novel sound variants; and (iii) 4 recombined sequences, such that D (not B) followed A and C followed B (with an X element between each one in the tri-element condition). See below for full details of how these sequences were defined and constructed. A 5 s response window was left between each bi- or tri-element playback sequence, beginning from the onset of the final sound in the sequence, during which we coded for behavioural responses to the playback trial (latency of first look towards the speaker, and total gaze duration). The test phase was carried out in a featureless 200 × 400 cm room, except for the remotely operated Sony HDR-CX 240E digital camcorder and speaker (volume set at 55 dB to mirror natural call volume) positioned together on a tripod 100 cm from the mesh front of the box containing the bird (the same box they roosted in overnight), inside which the bird could move freely. The speaker was not concealed, so that the source of the sound would be immediately apparent to the subject. The experimenter operated the equipment remotely from outside the room so that they were not visible to the bird.

#### Acoustic stimuli details

##### Acoustic elements

We generated 6 types of acoustic elements, which we refer to as elements A, B, C, D, Xi and Xii (Figure 2). For each element type, we generated 16 pitch variants, each of which had identical pitch contours but different starting pitches, which we refer to as e.g. A1, A5, B3, C16, etc. Half of these variants (numbers 1–8) were used in familiarisation sequences, with variant 1 having its onset start at 500hz and each subsequent variant starting 50Hz higher. Variants 9–16 were used in the pitch-shift and recombination sequences, with variant 9 starting at 1100Hz and each subsequent variant starting 50Hz higher. A gap of 250Hz (850–1100) was inserted between variants 8 and 9 to increase the saliency of the difference between familiarisation and pitch-shift/recombination sequences. All elements within a sequence were played back at the same volume in an otherwise silent room. All elements were well within the babbler’s natural call range (<300 to >4000 Hz) with previous research suggesting that babblers can discern natural call elements that differ in frequency of <50 Hz.^37^ All elements had a duration of 1500ms, with a 10ms volume fade in/out to eliminate sound onset effects. All stimuli were generated using the software Praat.^58^ These elements were designed with the intent that each category is easily acoustically distinguishable from the next, and have been productively applied to examining the sequential processing abilities of humans and primates (*32*). A sample sound generation script, and all sounds associated with this experiment, can be downloaded from: https://osf.io/mhgcx/.

##### Sequence composition

The birds assigned to the bi-element condition were exposed to sequences comprised of elements A, B, C and D only, while those exposed to the tri-element condition additionally heard Xi and Xii elements. The reason for oscillating between the use of these two X elements was to minimise the likelihood that individuals simply learned another set of associations between the middle (i.e. Xi) and edge elements (i.e. A and B) to process the tri-element sequences, thus necessitating an understanding of the relationship between the non-consecutive first and last elements. In other words, while the relationship between A/B or C/D had total predictive certainty, the predictive accuracy between edge and central elements was relatively low, encouraging reliance on these non-adjacent relations. An additional consideration was that introducing variability to this central element mitigated the possibility that the birds would ignore it and parse the edge elements as adjacent. Within both bi- and tri-element sequences, there was 500ms of silence between each element. Correspondingly, bi-element sequences lasted a total of 3500ms, whereas tri-element sequences lasted 5500ms.

Familiarisation sequences were random assortments of frequency variants 1–8 played during the familiarisation phase (i.e. starting frequencies varying from 500 to 850 Hz). Pitch-shifted sequences were identical to familiarisation sequences in all regards except that they were comprised of variants 9–16 (i.e. starting frequencies from 1100-1450 Hz). Finally, recombined sequences were also pitch-shifted (i.e. from 1100-1450 Hz), but this time the sequences were recombined in the form A(Xi/ii)D and B(Xi/ii)C.

##### List composition

In the familiarisation phase, sequences were played in a pseudorandom order such that each combination of element variants (e.g., A1-B6, A2-B4, C7-D2, A5-B5 etc; total combinations = 60) appeared four times (240 sequences in the list), but never more than three times in a row. All lists contained the same number (N = 120) of AB sequences and C-D sequences (with Xi/Xii inserted in the tri-element condition).

During the test phase, each bird was exposed to 4 familiar, 4 pitch-shifted and 4 recombined sequences (12 trials total). These were played in pseudorandom order such that no condition was heard more than three times in a row to minimise habituation. Whether a subject heard a pitch-shifted or recombination sequence as the first playback in the list was counter-balanced across subjects, so that a potential effect of the mere novelty of the pitch-variants was controlled for. For familiar and pitch-shifted sequences in the test phase, half were A-B sequences, and the other half were C-D sequences. For the recombined sequences, half were of form A-D and the other half were C-B (with Xi/Xii inserted in the middle for sequences in the tri-element condition). Sequences heard by birds in the bi- and tri-element conditions were identical in the sounds used to comprise them, the only difference being the addition of Xi and Xii in the middle of tri-element sequences.

### Quantification and statistical analysis

All videos were coded frame-by-frame using BORIS behavioural observation software^59^ by SKW. Specifically, we coded the time and duration of each occasion the bird looked directly towards the speaker during the 5 s response window that followed the onset of the final sound in a playback sequence. A direct look was determined as occasions where the bird’s beak was oriented straight-on with the camera (which was positioned at the speaker). This response measure has been validated in previous playback experiments with this species.^8^ While it may be that some birds attended to the speaker at an offset angle, it was not possible to reliably code a standardised offset criterion (e.g. orientations within 30 degrees of the speaker) due to the non-uniform positionality of the birds within the cage during testing. If the bird was already looking towards the camera at the time of onset of the final element in a sequence, or if the bird was vocalising over the playback sounds, the trial was not used for analysis (55 / 252 trials). When not reacting to the experiment, birds were generally resting or moving inside the box. For each trial, we then extracted: (a) the latency of a subject’s first look towards the speaker (hereafter ‘response latency’); and (b) the total amount of time spent looking directly toward the speaker (hereafter ‘gaze duration’).

Inter-observer reliability tests were carried out on ∼25% of all trials (N = 50, N = 25 for each condition). To this end, a second coder (J.G.M.) was provided with muted clips of the 5 s response windows to ensure that they were blind to both condition and stimulus type (the primary coder, SKW, used unmuted videos). Pearson’s correlation analysis suggested an overall high level of agreement between observers for both response latency (bi-element condition r: 0.937, tri-element condition r: 0.966, overall r: 0.952). and gaze duration (bi-element condition r: 0.804, tri-element condition r: 0.952, overall r: 0.841).

To allow for the inclusion of trials in which there was no response from the subject, we censored this data using survival analyses.^60^ Specifically, we employed Bayesian Cox proportional hazards models.^60^ One full model and one null model were fit for each condition (bi-element and tri-element conditions) and each outcome variable (response latency and gaze duration) for a total of 8 models. The full model included a fixed effect of sequence type (3-level factor: familiarisation, pitch-shifted and recombination sequences) and a random effect of individual identity, whereas the null model contained only the latter. Comparisons between full and null models were carried out using Watanabe-Akaike information criterion (wAIC) weights in order to determine the relative likelihood that a given model provided the best predictive fit for the data. Whether there was a difference between conditions (bi-element / tri-element) in response to the same sequence type was then explored using six further models examining each combination of sequence-type and outcome variable. Because multiple datapoints were used for each subject, each of these models also included random intercepts for each individual.

All models were implemented in R^61^ and RStudio^62^ using the package ‘brms’.^63^ Model chain convergence was assessed by inspecting trace plots, rhat values (all equal to 1.00) and effective sample sizes (all over 1000). All data and code used for analysis, as well as markdowns of model outputs, can be accessed at the following repository: https://osf.io/mhgcx/.

## Data Availability

• All raw data used for analysis in this study have been deposited at the following Open Science Framework repository: https://osf.io/mhgcx/. This data is publicly available as of the date of publication. DOI is listed in the key resources table. • All original code has been deposited at the following Open Science Framework repository: https://osf.io/mhgcx/. DOI is listed in the key resources table. • Any additional information required to reanalyze the data reported in this paper is available from the lead contact upon request.
