# Supplementary material for: Cognitive constraints on vocal combinatoriality in a social bird
Source: iScience. 2023 May 26;26(7):106977. doi: 10.1016/j.isci.2023.106977 (PMC10275715; doi:10.1016/j.isci.2023.106977)
Supplement: Document S1. Figure S1 and Table S1 [file mmc1.pdf]

iScience, Volume *26*

Supplemental information

Cognitive constraints on vocal combinatoriality in a social bird

Stuart K. Watson, Joseph G. Mine, Louis G. O'Neill, Jutta L. Mueller, Andrew F. Russell, and Simon W. Townsend

Supplemental material

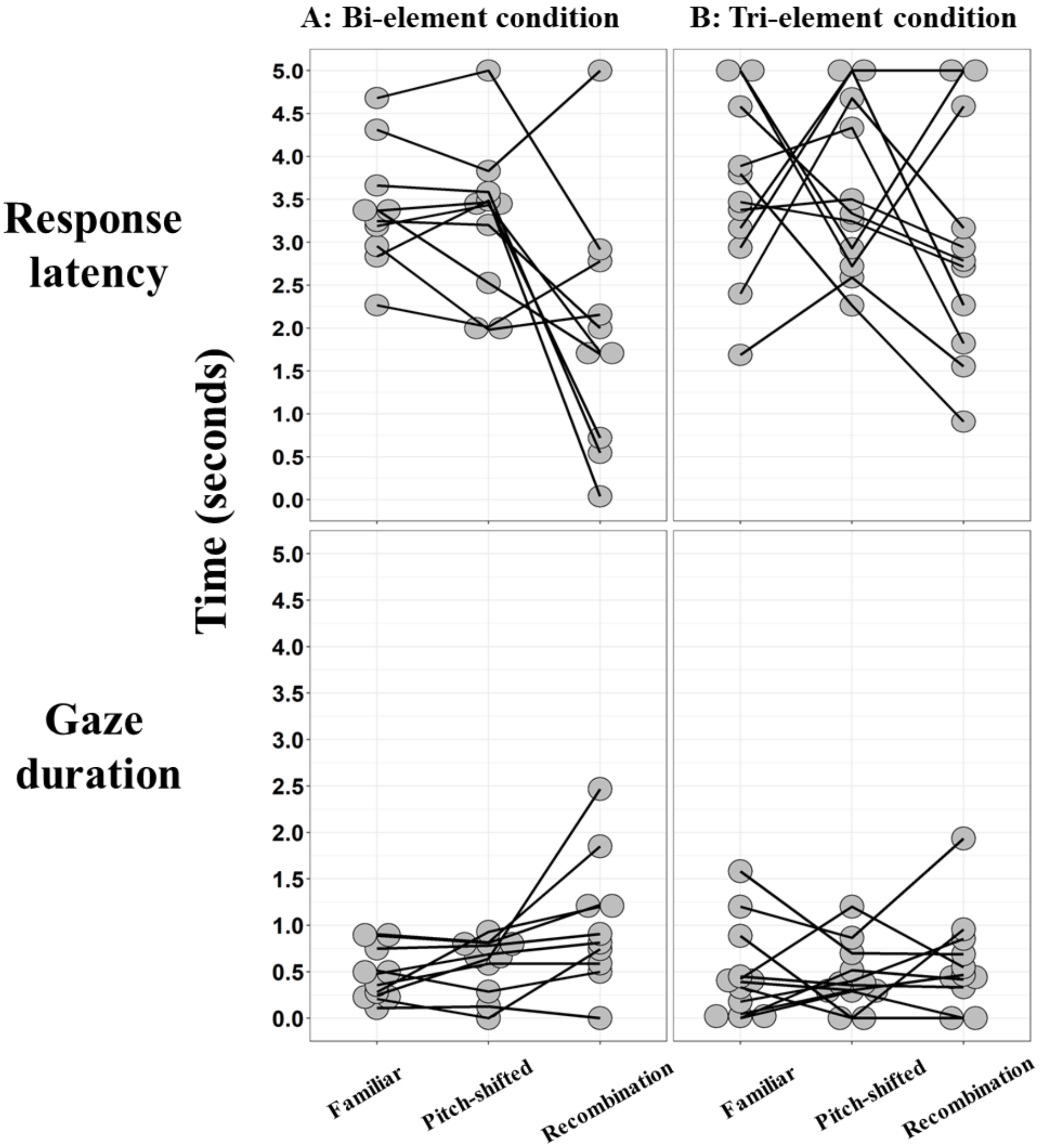

Figure S1. Descriptive plots for each individual in each condition, related to Figure 3. Mean response measures (Top: Response latency, Bottom: Gaze duration) for each subject (circles), in each condition (A: Bi-element, B: Tri-element) and each sequence type. Black lines connect datapoints coming from the same individual. Latency of 5s indicates that the bird did not react within the response window.

Table S1. Descriptive summary statistics for each response measure and condition. Related to Figure 3.

Condition	Response measure	Stimuli	Mean (seconds)	Standard deviation	N trials	N subjects
Bi-element	Response latency	Familiar	2.13	1.95	35	10
		Pitch-shifted	3.45	1.66	37	10
		Recombination	3.21	1.55	39	10
Bi-element	Gaze duration	Familiar	1.04	1.08	35	10
		Pitch-shifted	0.47	0.65	37	10
		Recombination	0.58	0.65	39	10
Tri-element	Response latency	Familiar	2.97	2.12	39	12
		Pitch-shifted	3.53	1.76	36	12
		Recombination	3.58	1.84	33	12
Tri-element	Gaze duration	Familiar	0.59	0.85	39	12
		Pitch-shifted	0.45	0.65	36	12
		Recombination	0.47	0.76	33	12
